# Defensive functions provoke similar psychophysiological reactions in reaching and comfort spaces

**DOI:** 10.1038/s41598-021-83988-2

**Published:** 2021-03-04

**Authors:** G. Ruggiero, M. Rapuano, A. Cartaud, Y. Coello, T. Iachini

**Affiliations:** 1 Laboratory of Cognitive Science and Immersive Virtual Reality, CS-IVR, Department of Psychology, University of Campania L. Vanvitelli, Viale Ellittico, 31, 81100 Caserta, Italy; 2 UMR 9193 - SCALab - Sciences Cognitives et Sciences Affectives, CNRS, CHU Lille, University of Lille, 59000 Lille, France

**Keywords:** Neuroscience, Psychology

## Abstract

The space around the body crucially serves a variety of functions, first and foremost, preserving one’s own safety and avoiding injury. Recent research has shown that emotional information, in particular threatening facial expressions, affects the regulation of peripersonal-reaching space (PPS, for action with objects) and interpersonal-comfort space (IPS, for social interaction). Here we explored if emotional facial expressions may similarly or differently affect both spaces in terms of psychophysiological reactions (cardiac inter-beat intervals: IBIs, i.e. inverse of heart rate; Skin Conductance Response amplitude: SCR amplitude) and spatial distance. Through Immersive Virtual Reality technology, participants determined reaching-distance (PPS) and comfort-distance (IPS) from virtual confederates exhibiting happy/angry/neutral facial expressions while being approached by them. During these interactions, spatial distance and psychophysiological reactions were recorded. Results revealed that when interacting with angry virtual confederates the distance increased similarly in both comfort-social and reaching-action spaces. Moreover, interacting with virtual confederates exhibiting angry rather than happy or neutral expressions provoked similar psychophysiological activations (SCR amplitude, IBIs) in both spaces. Regression analyses showed that psychophysiological activations, particularly SCR amplitude in response to virtual confederates approaching with angry expressions, were able to predict the increase of PPS and IPS. These findings suggest that self-protection functions could be the expression of a common defensive mechanism shared by social and action spaces.

## Introduction

The space surrounding our body is of primary importance for survival needs. Indeed, we automatically monitor any animate or inanimate stimulus that can potentially enter the margins of the body space. In social psychology, the interpersonal space (IPS) is the area that individuals keep around themselves where others cannot enter without causing discomfort^1,2^. A typical task adopted to evaluate the size of IPS is based on comfort-distance judgments (‘stop-distance’ paradigm^3^: participants have to stop the encounter at the point where they still feel comfortable with the other’s proximity^2,4–7^. Longstanding research has demonstrated that this space increases in uncomfortable/threatening situations and decreases in comfortable/safe situations^8–15^.

In the neuro-cognitive domain, the peripersonal space (PPS) is the multisensory area around our body where physical interactions between the individual and the environment/objects can take place (e.g.^8–11,16–23^. Much evidence has demonstrated that PPS is represented by highly integrated multisensory and motor processes in fronto-parietal and posteromedial areas^11,19–23^. PPS is also commonly used to define the portion of space within the reach of our limbs^11,24,25^. Here we are referring to the portion of reachable space and we adopt the reachability judgment to measure its size: participants have to judge whether objects or confederates are reachable or not^9,13,26–28^. Importantly, PPS is also thought as “a margin of safety” involved in defensive functions^13,16–18,29^.

Much evidence has shown that socio-emotional information can modulate the PPS (for reviews see^30–32^). For example, Teneggi et al.^33^ reported that the boundaries of PPS shrank in the presence of another person as compared to a mannequin. The size of PPS is also modulated by dangerous objects that may threaten physical integrity^4,9,16,26,34,35^. Moreover, subcortical defensive responses such as the hand-blink reflex are regulated by the type of social interaction and threat^36^.

According to an ‘action-centered’ perspective, Lloyd^31^ proposed a ‘cognitive intentional’ route in which human social and spatial interactions would be mediated by visuo-spatial, motivational-affective and cognitive-behavioural factors. The same underlying processes would mediate interactions with both inanimate and animate objects, with links to socio-emotional and motivational systems encoding the relevance of those interactions. Coherently, a recent fMRI study has shown that intrusions into personal space caused by looming social stimuli (i.e., faces) activate cerebral areas (e.g. dorsal intraparietal sulcus and ventral premotor cortex) that are similarly involved in the representation of PPS^37^. Therefore, the space around the body can be seen as the physical space where some social actions occur on the basis of their emotional and motivational relevance^31^. This proposal integrates classic proxemics models of IPS with neuroscientific models of PPS. In fact, even in proxemics studies a perceived socio-emotional stimulus (like a threatening person or a stressing situation) can be one of the most relevant factors in regulating the equilibrium between interpersonal distance and social interaction^4,6,38,39^. Moreover, in line with Patterson’s arousal model of intimacy exchange^40^ and Middelmist and Knowels’ arousal model of personal space invasions^41^, positive (happy) or negative (angry) connotations of non-verbal social interactions may provoke different activations of the arousal and behavioral modulations.

In sum, the interrelation between the peripersonal-reaching space and the interpersonal-comfort space may lie in their protective functions along with their sensitivity to socio-emotional information (e.g.^14,15,42–46^). Recently, Ruggiero et al.^43^ proposed that these spaces share a similar sensitivity particularly in the presence of threatening signals. Using Immersive Virtual Reality (IVR) technology, they compared reaching-distance (distance at which people perceive a stimulus as reachable, for PPS) and comfort-distance (distance that people prefer from other persons, for IPS) in relation to virtual humans exhibiting angry/happy/neutral facial expressions. The results revealed that when participants were approached by virtual confederates with angry facial expressions both IPS and PPS increased.

Therefore, if IPS and PPS share a similar protective function, we can argue that behavioural and psychophysiological reactions should show a similar modulation in response to threatening stimuli. To this end, we devised an experimental paradigm based on the combination of facial emotional expressions (happy, angry, neutral) and an invasive spatial approach to capture psychophysiological and behavioral responses. While participants were approached by the virtual confederates and determined their reaching and comfort distance, psychophysiological reactions in terms of the skin conductance response (SCR, i.e., amplitude of phasic change in electrical conductivity of skin;^47–49^) and cardiac inter-beat intervals (IBIs; inverse of heart rate)^50^ were acquired.

The inter-beat interval represents the length of time between consecutive heartbeats, regulated by the sympathetic and parasympathetic branches of the Autonomic Nervous System (ANS). The ANS regulates physiological signals such as muscle tension, respiration, facial expressions, pupillary changes, articulation, tone of voice, posture, gesture, skin temperature or activity of sweat glands^40^. Therefore, faster heart rates correspond to shorter inter-beat intervals and vice versa. Electrodermal activity (EDA) is composed of two components: the tonic EDA (i.e. Skin conductance level, SCL) and the phasic skin conductance response (SCR)^51^. While the SCL reflects general changes in autonomic arousal representing the baseline level of the signal, the skin conductance response (SCR) is associated with phasic sympathetic nervous discharges^35^ and represents the direct response to a specific stimulus^51^. Moreover, it is known that the amplitude of SCR increases linearly with the increase of the intensity of emotional stimuli compared to neutral ones^52–54^. Therefore, due to these specific characteristics, we considered the SCR as a more informative measure of emotional arousal albeit recognizing that literature suggests both SCL and SCR are important and may rely on different neural mechanisms^47,55^.

Previous studies used different psychophysiological measures to understand the impact of socio-emotional stimuli in social interaction. For example, Leutgeb et al.^56^ found impaired social cognition in prisoners due to altered functional connectivity between cerebellum, amygdala and within the DLPFC. Llobera et al.^57^ reported an increased electrodermal activity when participants were approached by virtual characters at varying distances. Tajadura-Jimenez et al.^58^ observed that listening to music that induced positive emotions provoked a reduction in participants' personal space when approached by others. Studying the illusion of body ownership (i.e. treating a virtual body as if it were our real body), Petkova and Ehrsson^59^ found higher SCRs when such a fake body was threatened (see also^35,60,61^).

Building on this, we deem that the present study may contribute to clarify, in physiological terms, the role of the protective functions attributable to PPS and IPS during social interactions. More specifically, psychophysiological indexes can help explain the individual differences we normally observe in the size of PPS and IPS and, at the same time, can reveal bodily reactions that prepare for defensive behaviours. Using the IVR as in Ruggiero et al.^43^, participants determined reaching-distance and comfort-distance while being approached by virtual confederates exhibiting either an angry, happy or neutral (control) facial expression.

VR technology is being increasingly used in proxemics research (e.g.,^4,13,15,43,44,46,57,62^). VR enables experimenters to control the appearance and behavior of virtual humans and create physical transformations or threats that could not easily be implemented in the laboratory^63^ with a high degree of similarity to real life^6,63–68^. Despite some criticisms such as the lack of physical contact^69^ and possible perceptual alterations^65,70,71^, evidence has robustly shown that, proxemically, individuals treat virtual humans as if they were actual humans^4,13,15,43,44,46,57,62^. Therefore, VR is an optimal means to assess in an ecologically valid and controlled way the spatial behaviour of participants during social interactions and to accurately acquire the participants' psychophysiological responses.

We hypothesized that if IPS and PPS share a similar protection function, they should show similar sensitivity to emotional cues expressing threats. More precisely, angry faces should favour avoidant behaviours and thus larger distances, with concurrent higher SCR and shorter IBI values, than happy and neutral faces.

## Results

### Skin conductance response amplitude

**Figure 1:**
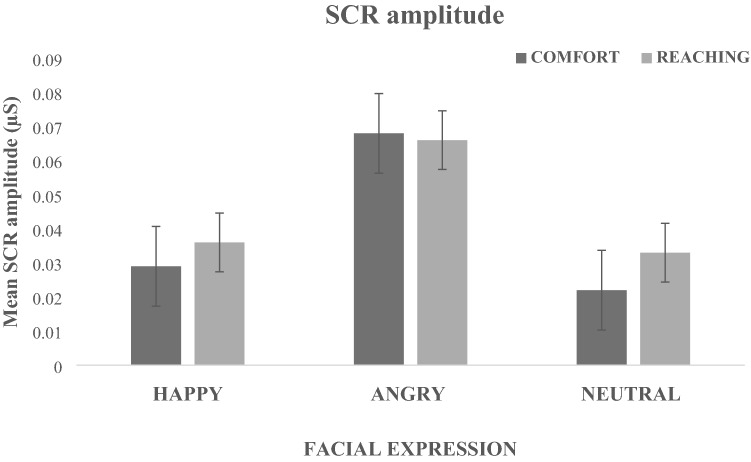
Effect of Facial expression on SCR amplitude. The graph shows the mean SCRs amplitude (μS) as a function of the three facial expressions (Happy-Angry-Neutral) in the comfort- and reaching-distance tasks. Error bars represent the standard error.

The ANOVA showed a significant main effect of Facial Expressions, F(2,46) = 11.413, *p* < 0.001, *η*^*2*^_*p*_ = 0.33), due to angry expressions inducing higher SCRs amplitude (M = 0.067 μS; SD = 0.07) than the other two facial expressions (Happy: M = 0.033 μS; SD = 0.03; Neutral: M = 0.027 μS; SD = 0.02; at least *p* < 0.001) (Fig. 1). Neither a main effect of the Task (F < 1) nor a significant Task x Facial Expressions (F < 1) appeared.

Finally, two separate one way ANOVAs comparing Facial Expressions vs SCRs-Baseline showed that in both Comfort (F(3,69) = 13.188, *p* < 0.001, *η*^*2*^_*p*_ = 0.36) and Reaching (F(3,69) = 19.521, *p* < 0.001, *η*^*2*^_*p*_ = 0.46) tasks the experimental conditions induced higher SCRs than Baseline (at least *p* < 0.05; with the exception of the neutral expression in the Comfort Task).

### Cardiac inter-beat interval (IBI)

**Figure 2:**
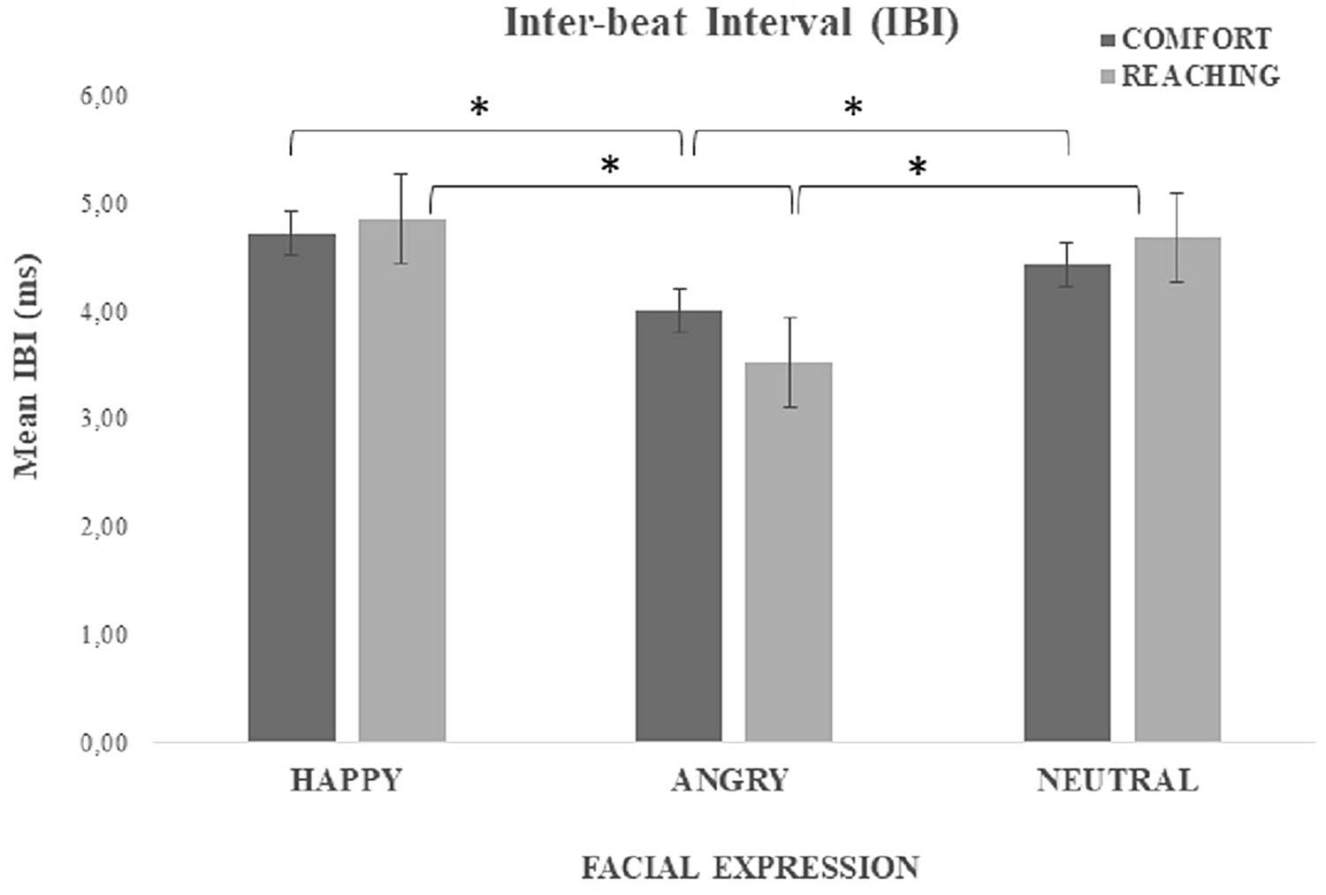
Effect of Facial expression on IBI values. The graph shows the mean IBIs (msec) as a function of the three facial expressions (Happy-Angry-Neutral) in the comfort- and reaching-distance tasks. Error bars represent the standard error.

The ANOVA showed a significant main effect of the Facial Expressions (F(2,46) = 36.833, *p* < 0.001, *η*^*2*^_*p*_ = 0.62. Post-hoc analysis showed that IBIs were shorter when dealing with angry (M = 3.77, SD = 0.63) rather than happy (M = 4.79, SD = 0.96) and neutral (M = 4.56, SD = 0.85) virtual confederates (at least *p* < 0.001). No significant effect of the Task factor appeared (F < 1). A significant Task x Facial Expressions interaction was found (F(2,46) = 5.778, *p* < 0.001, η^2^_p_ = 0.20. As shown in Fig. 2, IBIs in response to angry virtual confederates were shorter in the Reaching Task than in the other conditions. Post-hoc analysis revealed that within the Reaching Task, IBIs in the presence of angry confederates were significantly shorter than the other conditions (at least *p* < 0.001), apart from angry confederates in the Comfort Task. Finally, within the Comfort Task, IBIs were shorter when dealing with angry than happy, not neutral, virtual confederates (*p* < 0.01).

Finally, two separate one way ANOVAs comparing Facial Expressions vs IBIs-Baseline showed that in both Comfort (F(3,69) = 342.497, *p* < 0.001, *η*^*2*^_*p*_ = 0.94) and Reaching (F(3,69) = 282.199, *p* < 0.001, *η*^*2*^_*p*_ = 0.92) tasks the experimental conditions induced shorter IBIs than Baseline (at least *p* < 0.01).

### Spatial distance

**Figure 3:**
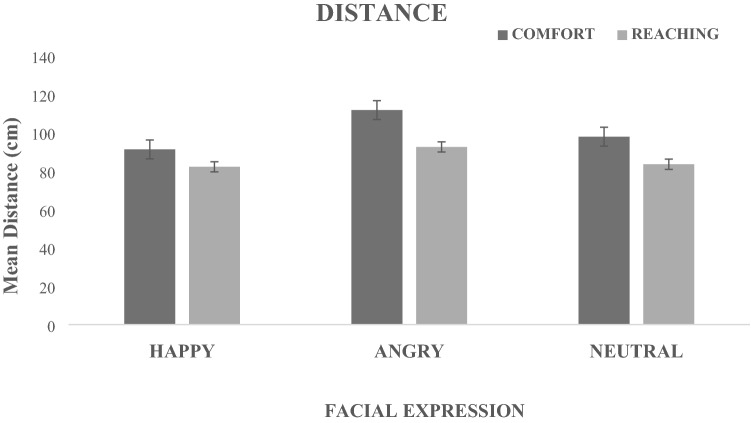
Effect of Facial expression on distance. The graph shows the mean distances (cm) as a function of the three facial expressions (Happy-Angry-Neutral) in the comfort and reaching distance tasks. Error bars represent the standard error.

The ANOVA showed only a significant main effect of Facial Expressions (F(2,46) = 17.086, *p* < 0.001, *η*^*2*^_*p*_ = 0.43). The Tukey HSD post-hoc test revealed that the effect was due to the angry expressions (M = 102.15 cm; SD = 41.33) which induced a larger expansion of the distance with respect to happy (M = 86.73 cm, SD = 39.29) and neutral (M = 90.72 cm; SD = 38.17) expressions (at least *p* < 0.001) (Fig. 3). Although the Task factor did not reach significance, (F(1,23) = 3.260, *p* = 0.08, the Comfort distance (M = 100.30 cm, SD = 43.44) was larger than the Reaching distance (M = 86.09 cm, SD = 35.74). Finally, no significant Task x Facial Expressions interaction emerged (F(2,46) = 2.358, *p* > 0.05).

### Sex differences

The ANOVA showed a main effect of Virtual Confederate's sex (F(1,22) = 74.171, *p* < 0.0001, *η*^*2*^_*p*_ = 0.77). Distance was larger from male (M = 100.91, SD = 43.17) than female (M = 85.483, SD = 38.51) virtual confederates. Virtual Confederate's sex interacted with Task, F(1,22) = 5.787, *p* < 0.05, *η*^*2*^_*p*_ = 0.21. The interaction was due to the Comfort Task, where distance from virtual male confederates was larger than all other conditions (at least *p* < 0.001). Moreover, within each Task, distance was larger with male than female virtual confederates (*p* < 0.05). There emerged neither main effect of Participant’s sex (F(1,22) = 1.398, *p* = 0.25) nor main effect of Task (F(1,22) = 3.017, *p* = 0.09) nor their interaction (F < 1). Finally, neither Virtual Confederate's sex X Participant’s sex interaction (F < 1) nor 3-way interaction (F < 1) were found.

### Regression analysis

**Figure 4:**
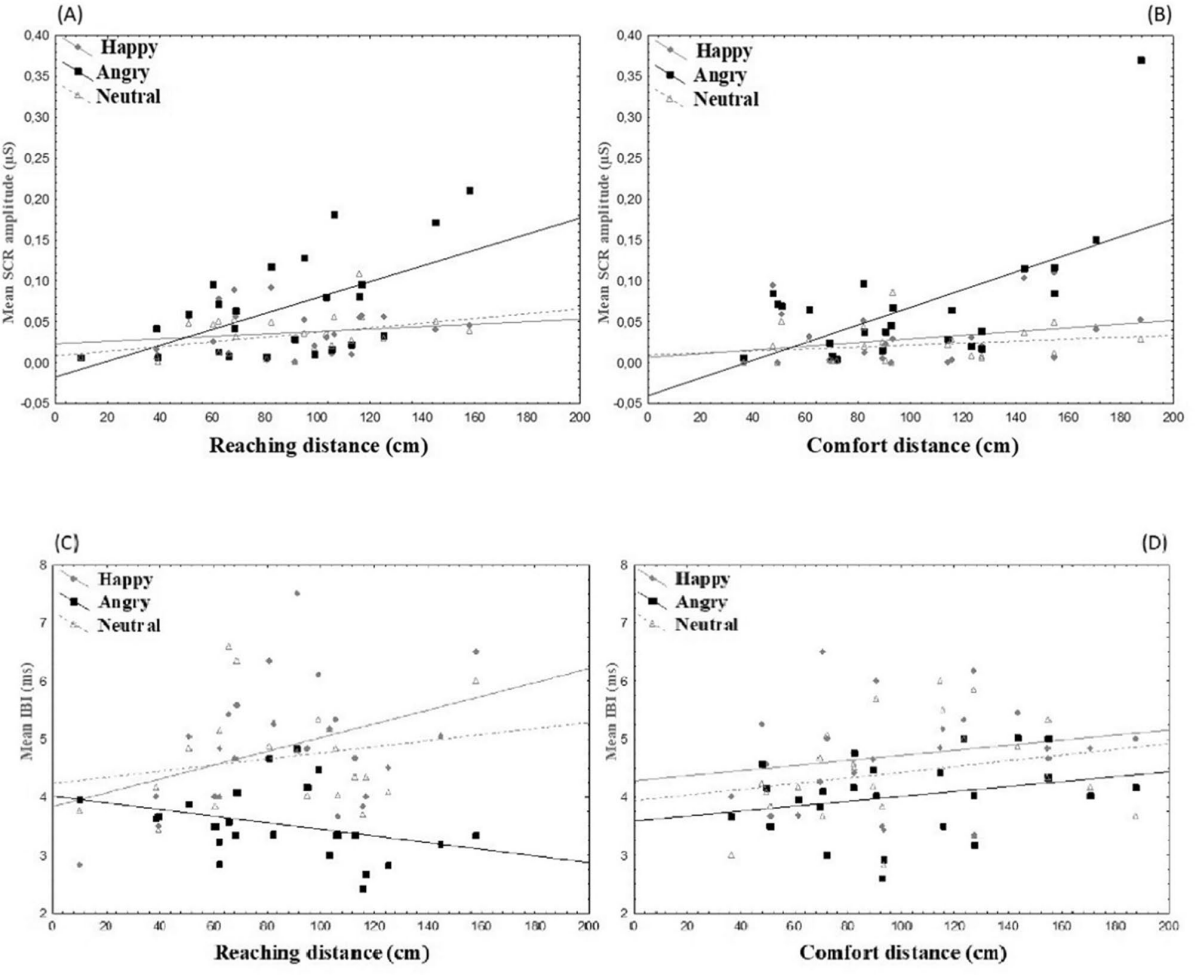
Scatterplots about the relationship between psychophysiological indexes and mean spatial distances. *Top*, the graphs show SCR amplitude values (μS) for happy, angry and neutral facial expressions (ordinate) and mean Reaching (cm; *panel A*) and Comfort (cm; *panel B*) distances (abscissa); *bottom*, the graphs show the Inter-Beat Interval (ms) values for the happy, angry and neutral facial expressions (ordinate) and the mean Reaching (cm; *panel C*) and Comfort (cm; *panel D*) distances (abscissa).

The multiple regression analysis allowed us to examine how much of the variance in the distance regulation was explained by the psychophysiological indexes. In regards to the Reaching Task, the whole model was significant: F (6, 17) = 9.476, *p* < 0.001, R^2^ = 0.77, R = 0.88. However, only three predictors contributed significantly to the model: amplitude related to angry confederates (t = 2.884, *p* < 0.05, B = 246.999, SE 85.657, Beta = 0.42); IBI related to happy confederates (t = 4.788, *p* < 0.001, B = 30.402, SE 6.349, Beta = 0.92) and angry confederates (t =  − 3.754, *p* < 0.005, B = − 37.682, SE 10.037, Beta = − 0.65). As the SCR amplitude increased in reaction to angry confederates, the distance also increased. The shorter the IBI in reaction to the happy confederates the shorter the distance, i.e. the faster the heartbeat in the presence of the happy confederates, the shorter the distance. Instead, the shorter the IBI in reaction to the angry confederates the larger the distance, i.e. the faster the heartbeat in the presence of the angry confederates, the larger the distance. Also in regard to the Comfort Task the whole model was significant, F (6, 17) = 2.804, *p* < 0.05, R^2^ = 0.50, R = 0.70. However, only one predictor gave a significant contribution to the model, that is amplitude in reaction to the angry confederates (t = 3.208, *p* < 0.01, B = 355.310, SE 110.758, Beta = 0.64). The larger the SCR amplitude in the presence of the angry confederates the larger the distance. Scatterplots depicting the relationship between the psychophysiological measures and the distances are shown in Fig. 4.

## Discussion

In this study IPS and PPS were compared to assess the influence of the emotional signals (i.e., facial expressions) on psychophysiological patterns, that is SCR amplitude and cardiac IBI. By measuring these two physiological indicators, we aimed to gain a better understanding of the extent to which comfort and reaching spaces showed a similar sensitivity to emotional stimuli attributable to basic defensive functions. Since defensive responses are mainly based on the early stages of processing, they can be better detected by psychophysiological measures^72^. These measures, complemented by behavioural data, can provide a particularly informative and reliable source of evidence.

Overall, the results showed a similar physiological activation in reaction to emotional facial expressions in IPS and PPS, although with different shades. Specifically, independently of the task, in both spaces SCR values increased during interaction with angry virtual confederates and decreased with happy and neutral ones^73^. Similarly, in both PPS and IPS the IBI values were shorter when participants were approached by angry virtual confederates than the others. However, the reaching task seemed more sensitive to emotional expressions, with particularly short IBIs in the presence of angry virtual confederates than other emotional expressions. In the comfort task, only the comparison between angry and happy emotional expressions was significant. Despite the differences, in both tasks the psychophysiological response was modulated mainly by the threatening stimulus, i.e. an expression of anger. This suggests that psychophysiological responses depended on the content of the emotional stimulus and the nature of the defensive mechanism^74^. These responses trigger avoidance behaviour when humans, as well as non-human species, are exposed to a threat that invades their margin of safety^75,76^. Consistently, the regression analyses showed that psychophysiological indexes affected the regulation of both interpersonal-comfort distance and peripersonal-reaching distance. Again, the two tasks were more similar when considering the SCR amplitude than the IBI. In fact, in both cases there was an enlargement of the distance as the SCR increased in the presence of angry confederates. In regards to IBI, the activations were finely modulated by the emotional content in the reaching task but not the comfort task. In fact, shorter IBIs in response to angry confederates predicted larger reaching distances, whereas shorter IBIs in response to happy confederates predicted shorter reaching distances. Therefore, when we have to assess whether we can touch a person, the acceleration of the heartbeat in response to a happy expression leads to a reduction in distance, while the same acceleration in response to an angry expression leads to an increase in distance.

The whole results suggest that the modulation of skin conductance in response to threatening stimuli may represent a consistent point of contact between PPS and IPS and confirm a shared defensive function. Thus, they are in line with an evolutionary perspective in which avoidance mechanisms are among the most important biological adaptations that have evolved to ensure the survival of the organism^77–81^. Instead, when other persons communicate positive feelings (such as happy facial expressions), individuals are less prone to defend their space and tend to facilitate the social interaction^49,82–84^. This suggests that avoidance reactions supported by psychophysiological responses reflect the optimal regulation of arousal and ensure an adequate self-protection barrier around us^1,2,26,34,38,77,85,86^.

Furthermore, the behavioural data were in line with previous literature. Indeed, both comfort and reaching distances were larger when interacting with angry virtual confederates compared to happy and neutral ones (e.g.^43^; see also^14^). Our results about sex-related effects are in line with proxemics literature showing that people prefer a larger distance from male than female confederates^2,3,44,87–89^. A comparison of the two tasks shows that the effect was particularly evident in the comfort task. In fact, the comfort distance to the male confederates was the widest of all, although the trend was similar in both tasks. This confirms that the comfort distance is particularly sensitive to socio-emotional information and that women are perceived as less threatening or potentially harmful than men^1,2,21,63,64,87^.

However, one problematic point needs to be addressed. There was no significant difference between comfort-distance and reaching-distance and therefore one may argue that the two tasks were not able to distinguish PPS and IPS. However, the metric difference between the two tasks was 14.21 cm (with the comfort distance being larger). This is in line with previous literature showing metric differences ranging from 4.4 cm to 25.2 cm^15,90^. The analysis of this literature suggests that the two tasks are differently sensitive to manipulated variables and this is expressed more in significant interactions than in a main effect of the task. In our study, the comfort distance from virtual males was larger than all other conditions; furthermore, the results about IBI data showed a stronger sensitivity to facial expressions in the reaching than comfort task. Consistently, the IBI psychophysiological indexes had a clear impact on reaching distance but no significant effect on comfort distance.

What do these results tell us about the nature of the space around the body? There is much debate on this issue, on the tasks that best address that nature and the terms that best describe it^29,91^. Here, our main purpose was to clarify the role of the protective functions attributable to these spaces during social interactions. The reaching space (imagine reaching the other) and the comfort space (tolerance of other's proximity) that we used in this study can be different per se. However, we found a point of contact when stimuli hinted at a potential threat that elicited the defensive function. This commonality suggests a close interaction between defensive function, representation of reaching space and comfort space, in line with an intriguing evolutionary account that considers human personal space as a safety buffer similar to the "flight zone" of animals^17,77,92^.

In conclusion, the current study showed an increase in SCR amplitude, a decrease in IBIs, and a widening of distance in response to threatening stimuli in both tasks. These convergent data support the hypothesis that the two spaces share a common defensive function^17,23,29,32,93–97^ and are also crosswise in line with the classic proxemics^4,38^ and modern action-centred^31,72^ models. An approaching threat, such as an angry face, provoked a rapid and automatic avoidance mechanism supported by psychophysiological responses and expressed in larger comfort and reaching distances. This mechanism is so fundamental for our defence that it constitutes a basic function of distance regulation^77^. Along an ideal continuum, the peripersonal reaching space should be more sensitive to the immediacy of physical contact, while the interpersonal comfort space should represent a socio-emotional mechanism of pre-alarm against a “potential” violation of personal space^13,40,41,57^. In turn, these fundamental defensive reactions could give rise to various social manifestations^77,78,94^. Therefore, the protective function of the representation of space around the body could be one of the most basic ways in which defensive physical actions intertwine with defensive social behaviours.

## Method

### Participants

Thirty-six participants were enrolled for the study in exchange for course credit. However, given the low quality of the recording of psychophysiological activities only 24 participants (11 women), aged 20–30 years (M_age_ = 24; SD = 2.2), were included in the present study. We also carried out a Power analysis to determine the required sample size for our within-subject design (G*Power 3.1.9.4;^98^). The required sample size was 24 with the following parameters: effect size = 0.25, power = 0.90, α = 0.05, and considering 6 repetitions by each emotion condition (6 trials with male virtual confederates and 6 trials with female virtual confederates). Participants had normal or corrected-to-normal vision, nobody claimed discomfort or vertigo during the IVR experience and reported being aware of the experimental purpose. All participants gave their informed consent to take part in the study and accepted to abstain from nicotine, caffeine and alcohol at least 3–4 h before the experimental session. Recruitment and testing were in conformity with the local Ethics Committee requirements of the Department of Psychology of the University of Campania L. Vanvitelli (Prot. n°151549/#8) and the subjects' consent was obtained according to the Declaration of Helsinki (1991; p. 1194).

### Setting and apparatus

The experimental setting and the virtual scenario were similar to those of previous studies^13,43^. The IVR equipment was installed in a 5 × 4 × 3 m room of the Laboratory of Cognitive Science and Immersive Virtual Reality (CS-IVR, Dept. Psychology). The equipment included the 3-D Vizard Virtual Reality Software Toolkit 4.10 (Worldviz, LLC, USA) with the Oculus Rift DK 2 head-mounted display (HMD) having two OLED displays for stereoscopic depth (images = 1920 × 1080; refresh rate 75 Hz). The IVR system continuously tracked and recorded participant’s position (sample rate = 18 Hz) through a marker on the HMD. Head orientation was tracked by a three-axis orientation sensor (Sensor Bus USB Control-Unit, USA). Visual information was updated in real time.

### Virtual scenario and virtual stimuli

**Figure 5:**
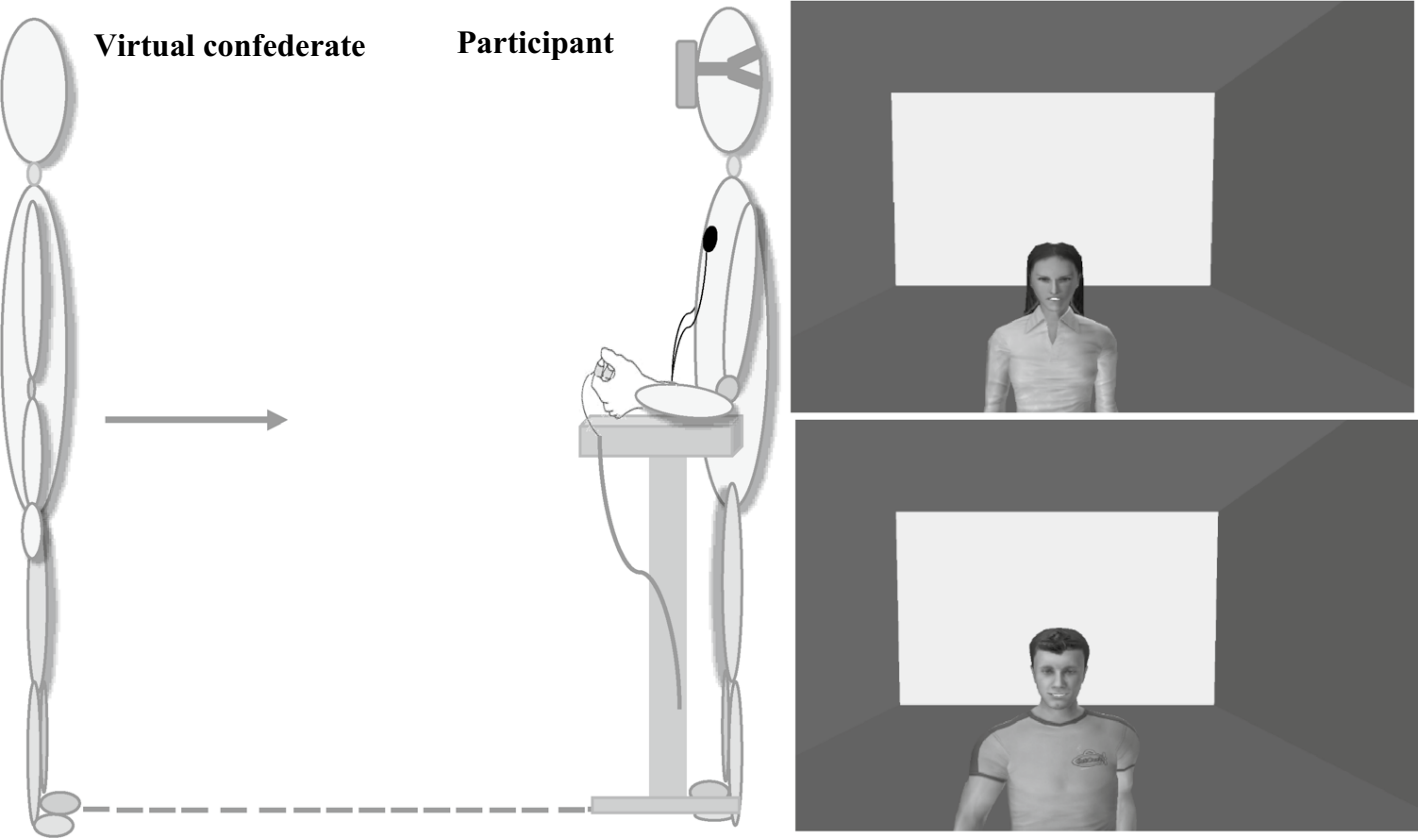
Example of experimental procedure and virtual stimuli. *On the left*, the panel shows a virtual confederate frontally approaching a participant. Participants, who were wearing HMD and psychophysiological devices, were told to stop virtual confederates when they thought they could reach them (Reaching-distance) or felt comfortable with their proximity (Comfort-distance). During the task, SCR, IBI and spatial distance were recorded. The small table was used to support participants’ forearm and non-dominant hand. The small table was on the left side of the participant. On the participant's chest were three ECG sensors for IBI acquisition; on the non-dominant hand were the electrodes for the SC acquisition. *On the right*, the panel shows examples of angry (*top*) and happy (*bottom*) facial expressions of female and male virtual confederates.

The virtual room consisted of green walls, white ceiling and grey floor (3 × 2.4 × 3 m). A total of twelve young confederates (six females) with neutral expression were selected among a colony of highly realistic virtual humans and were used for the present study (Vizard Complete Characters, WorldViz; USA). Virtual humans represented male and female adults aged about thirty years, wearing similar casual clothes and perceived as representation of Italian citizens (see again Fig. 1; on this point see^13,44^. Their height was 175 cm (males) and 165 cm (females). Their gaze was kept looking straight ahead throughout the trials^6^. Facial emotional expressiveness was obtained by modelling the virtual faces with 3DS Max (Autodesk) following the KDEF free-database (Karolinska Directed Emotional Faces;^99^). These virtual emotional confederates have been already rated, selected and used in a previous work^43^. The virtual confederates with the neutral emotional expressions represented the control condition for the positive and negative conditions. Therefore, the twelve virtual confederates showed the following facial expressions: happy (two males and two females), angry (two males and two females) and neutral (two males and two females) (see Fig. 5). Participants stood still and saw a virtual stimulus walking towards them at a constant speed (0.5 m^−1^). In both conditions the path between participants and stimuli was 3 m long. Walking movements of human avatars reproduced the natural swing of biological motion. In post-experimental debriefing, participants reported they clearly identified virtual confederates and their facial expressions as if they were ‘‘realistic persons’’.

### Psychophysiological measures

Psychophysiological signals (ECG and SC) were measured, amplified and recorded using *ProComp Infiniti,* (Thought Technology), a physiological monitoring device that encodes physiological signals in real-time, in separate channels and in parallel. An accompanying biofeedback software application, *Biograph Infiniti*, allowed the sampling and storage of the physiological data. Electrocardiogram (ECG) and Skin Conductance (SC) signals were obtained. ECG was measured by an EKG-Flex/Pro sensor with Triode electrodes snapped on, placed on the participant’s chest and abdomen. The sensor detects and amplifies the small electrical voltage that is generated by the heart muscle when it contracts. SC signals were acquired by 6-mm Ag/Ag–Cl electrodes placed on the palmar surface of the index and middle fingers’ distal phalanges of the non-dominant hand. A tiny electrical voltage is applied through the electrodes in order to establish an electric circuit where the subject becomes a variable resistor. The real-time variation in conductance is calculated. The electrodes were cleaned with an alcohol wipe between participants. All psychophysiological data were digitalized and stored at 2018 samples per second.

### Data treatment

Thanks to python language programming, we synchronised the IVR system with Procomp Infiniti and Biograph Infiniti software. This synchronization allowed us to acquire and extract behavioural and both psychophysiological measures. The ECG signals were band-pass filtered by ARTiiFACT^100^, a software tool for processing electrocardiogram data. IBIs were obtained with this software by extracting R-peaks from digitized ECG data and with a global threshold detection criterion. They were then corrected for physiologically impossible readings and artefacts. Afterwards, the average values of IBIs (ms) were calculated for each trial. SC signals were subsequently computed off-line by converting the digitized raw signals to skin conductance values by Ledalab, a Matlab-based software (MATLAB R2009b, The MathWorks Inc., Natick, MA, 2000) and then smoothed using the gauss-method. Ledalab provided a decomposition of skin conductance data into its tonic and phasic components. The SCR was used as index of specific event-related phasic changes calculated as the above threshold (0.01 mS) phasic activity within the response window. Here, the SCR amplitude was taken into account as the major metric of interest for the analysis. The SCR amplitude (i.e. the mean value only computed across those trials on which a measurable nonzero response occurred) was calculated for all significant (above threshold) SCRs within the response window, reconvolved from corresponding phasic driver-peaks. Square root transformation was conducted on raw SCR to normalize the data distribution. In addition, since we used an experimental paradigm triggering additive superposition of subsequent SCRs, the shape of a SCR could be altered by the trails of preceding phasic activity^101–104^. To overcome the problem, we applied a Continuous Decomposition Analysis (CDA) to better characterize the signal^105^. The CDA allows to decompose the skin conductance signal into its tonic and phasic (driver) data. Within the decomposition process, the optimization stage improves the estimation of the parameters of the impulse response function (IRF; representing basic SCR shape). When SCRs are overlapped, the SC signal process is modeled as a convolution process between the SudoMotor Nerve Activity (SMNA) and IRF under the hypothesis that EDA is controlled by SMNA resulting in a sequence of distinct impulses which regulate the eccrine sweat glands dynamics^105^. For each trial (10 sec), the trigger started as soon as the virtual stimulus appeared (time 0, onset). IBIs and SCRs data were extracted from the trigger to the end of the 8th sec. The sequence of the events was: 0 (trigger, onset stimulus), 2 sec (stationary stimulus), 4 sec (virtual stimulus move), 2 sec (the first two sec of blank).

### Procedure

Participants were introduced to the experimental room and led to a pre-marked position. They were given all instructions about the task and physiological recording equipment was also presented. Before placing all electrodes (for both SC and ECG measurements), the experimenter ensured that the attachment sites on the skin were dry. SC sensors were placed on the distal phalanges of the participant’s non-dominant hand. The ECG sensors were applied directly to the skin of the participant: two electrodes were positioned on the chest and one on the abdomen. In order to avoid discomfort, a female experimenter applied electrodes to women, a male experimenter applied electrodes to men. After placing the sensors, participants were invited to wear the HMD and to freely explore the virtual room with the gaze. Then, they were provided with a key-press device held in their dominant hand. Once familiarized with the IVR devices, the experimenter set up the recording equipment for the adaptation phase (15 min). After the adaptation phase, a baseline for each participant was recorded: 5 min-resting phase (empty room) and 20 sec (stationary virtual confederate with neutral expression). During baseline IBIs and SCRs values were acquired^106–108^. Next, a training session started: four virtual confederates (2 M/2F) exhibiting neutral facial expressions were exclusively used in the training phase. For each task (e.g. Comfort-distance), two virtual confederates (1 M and 1F) were randomly selected by the IVR software. Each virtual confederate appeared 4 times. Once the training phase was successfully completed, the testing phase began.

Each experimental session comprised six blocks administered in a counterbalanced order: positive facial expression, negative facial expression and neutral facial expression conditions, each repeated with comfort-distance and reaching-distance tasks. The comfort-distance instruction was: ‘‘Press the button as soon as the distance between you and the confederate makes you feel uncomfortable’’. The reaching-distance instruction was: ‘‘Press the button as soon as you can reach with your hand the confederate’’. Half of the participants started with the positive blocks, then the neutral blocks, finally the negative ones; the other half started with the negative blocks, afterwards the neutral blocks and then the positive ones. Throughout the experimental session, participants stood still and saw the virtual confederates walking towards them until they stopped them by button press. At the beginning of each comfort and reaching task, participants received a four-trial training session. The experimental flow included the task instructions (5 sec), a fixation cross (300 ms), and afterwards one virtual confederate appeared (3 m from the participant). For each block (positive, negative and neutral) and for each experimental task (two conditions: Comfort-Reaching), the IVR system selected two virtual confederates (one male and one female) showing happy, angry or neutral facial expressions (according to the emotional valence condition). Each virtual confederate appeared 3 times (either in comfort or reaching task conditions) resulting in 6 trials per block (tot. = 36 trials across all the six blocks). Each block started with 20 sec of rest period during which participants were immersed in the empty virtual room and no stimulation occurred. At the end of this time period, a virtual confederate (exhibiting happy, angry or neutral facial expression, according to the condition) was presented. The virtual confederate appeared (standing still for 2 sec) and then started moving toward the participants until the latter stopped the displacement by pressing the button (within 6 sec from onset). Afterwards, the virtual confederate disappeared and, after a blank (4 sec), another virtual stimulus was presented. The time-epoch target considered for both psychophysiological measures was from 0 up to 8 sec. Each experimental block (e.g., angry face + Reaching instructions) ended with a 10 sec rest period during which participants were again immersed within the empty virtual room and no stimulation occurred. As for the SCR, recommended recovery time (to the baseline value) is between 10 and 20 sec (e.g.^103,105,109^). For IBI data, the time spaces between heartbeats measure the variability of HR in ms, reflecting the flexibility of the cardiac function which accelerates rapidly when needed and returns rapidly back to the baseline pattern^110^.

Psychophysiological signals (IBIs, SCRs) were recorded for the entire duration of the experimental session and then extracted according to the trials and target time-epochs (0–8 sec; from stimulus onset). Each trial lasted a total of 10 sec. The sequence of the events was: 0 (trigger, onset stimulus), 2 sec (stationary stimulus), 4 sec (virtual stimulus move), 2 sec (the first two sec of blank), 2 sec (blank, not computed for the IBIs and SCRs). All participants gave the spatial distance judgment within the 6th sec (2 stationary + 4 virtual stimulus move). Next, participants remained immersed in the empty virtual room. In line with the literature, we considered as the first SCRs response from 1 to 3 sec after the onset of the stimulus^57–59^. We expected at least 2 peaks for each trial. The same temporal duration (0–8 sec) was considered when analyzing the IBI values^108^. Finally, at the end of the session, the experimenter removed the electrodes and the subjects were asked to evaluate their experience with the virtual confederates. They reported that they clearly identified their facial expressions as if they were ‘‘realistic persons’’.

### Data analysis

Three series of analyses were planned:

1. Three separate repeated measures ANOVAs with the Task (Reaching, Comfort) as a two-level factor and the Facial Expression (happy, angry, neutral) as a three-level factor were carried out on:
  - mean (μS) *Skin Conductance Response amplitude* (SCRs amplitude);
  - mean values (ms) of the *Cardiac Inter-Beat Interval* (IBIs);
  - mean participant-confederate distance (cm).

Furthermore, four repeated-measure one-way ANOVAs with 4 levels (Baseline vs happy, angry, neutral facial expressions) were performed on each Reaching and Comfort task for both SCRs and IBIs to compare experimental conditions and resting state (Baseline).

(2) In order to clarify the effect of sex on distance, a mixed design ANOVA with Participants’ sex as 2-level between factor and two-within factors, Virtual Confederate's sex (M, F) and Task (Reaching, Comfort), was performed on mean distances (cm).

To analyze all post-hoc effects, the Tukey HSD was used. The magnitude of significant effects was expressed by partial eta-squared (*η*^*2*^*p*). Data points outside M ± 2.5 SD were discarded (tot. number = 22).

(3) Furthermore, to assess the impact of the psychophysiological indexes on behavioural distances, we first evaluated the inter-item correlation between distances in relation to the three facial expressions within each task: Reaching task = 0.96; Comfort task = 0.89. Considering the strong inter-item correlation between distances, a mean Reaching distance and a mean Comfort distance were calculated and used as criteria for regression analyses. Therefore, to determine how the psychophysiological indexes in reaction to happy, angry and neutral confederates influenced the regulation of interpersonal-comfort distance and peripersonal-reaching distance, multiple regression analyses were carried out separately on each Reaching and Comfort Task with the three Amplitude scores and the three IBI scores as predictors and the mean Reaching and Comfort distances as criteria.
